# Characterization of an Indole-3-Acetamide Hydrolase from *Alcaligenes faecalis* subsp. *parafaecalis* and Its Application in Efficient Preparation of Both Enantiomers of Chiral Building Block 2,3-Dihydro-1,4-Benzodioxin-2-Carboxylic Acid

**DOI:** 10.1371/journal.pone.0159009

**Published:** 2016-07-08

**Authors:** Pradeep Mishra, Suneet Kaur, Amar Nath Sharma, Ravinder S. Jolly

**Affiliations:** Department of Bioorganic Chemistry, CSIR-Institute of Microbial Technology, Sector 39, Chandigarh, India; Montana State University, UNITED STATES

## Abstract

Both the enantiomers of 2,3-dihydro-1,4-benzodioxin-2-carboxylic acid are valuable chiral synthons for enantiospecific synthesis of therapeutic agents such as (*S*)-doxazosin mesylate, WB 4101, MKC 242, 2,3-dihydro-2-hydroxymethyl-1,4-benzodioxin, and N-[2,4-oxo-1,3-thiazolidin-3-yl]-2,3-dihydro-1,4-benzodioxin-2-carboxamide. Pharmaceutical applications require these enantiomers in optically pure form. However, currently available methods suffer from one drawback or other, such as low efficiency, uncommon and not so easily accessible chiral resolving agent and less than optimal enantiomeric purity. Our interest in finding a biocatalyst for efficient production of enantiomerically pure 2,3-dihydro-1,4-benzodioxin-2-carboxylic acid lead us to discover an amidase activity from *Alcaligenes faecalis* subsp. *parafaecalis*, which was able to kinetically resolve 2,3-dihydro-1,4-benzodioxin-2-carboxyamide with *E* value of >200. Thus, at about 50% conversion, (*R*)-2,3-dihydro-1,4-benzodioxin-2-carboxylic acid was produced in >99% e.e. The remaining amide had (*S*)-configuration and 99% e.e. The amide and acid were easily separated by aqueous (alkaline)-organic two phase extraction method. The same amidase was able to catalyse, albeit at much lower rate the hydrolysis of (*S*)-amide to (*S*)-acid without loss of e.e. The amidase activity was identified as indole-3-acetamide hydrolase (IaaH). IaaH is known to catalyse conversion of indole-3-acetamide (IAM) to indole-3-acetic acid (IAA), which is phytohormone of auxin class and is widespread among plants and bacteria that inhabit plant rhizosphere. IaaH exhibited high activity for 2,3-dihydro-1,4-benzodioxin-2-carboxamide, which was about 65% compared to its natural substrate, indole-3-acetamide. The natural substrate for IaaH indole-3-acetamide shared, at least in part a similar bicyclic structure with 2,3-dihydro-1,4-benzodioxin-2-carboxamide, which may account for high activity of enzyme towards this un-natural substrate. To the best of our knowledge this is the first application of IaaH in production of industrially important molecules.

## Introduction

Indole-3-acetamide hydrolase (IaaH) catalyses the conversion of indole-3-acetamide (IAM) to indole-3-acetic acid (IAA). IAA is phytohormone of auxin class and is widespread among plants and bacteria that inhabit plant rhizosphere. In plant-bacterial interactions, bacterial IAA is a lead molecule involved in pathogenesis [1] as well as phytostimulation [2]. Both the (***R***) and (***S***) enantiomers of 2,3-dihydro-1,4-benzodioxin-2-carboxylic acid and their corresponding amides are important building blocks in the synthesis of chiral therapeutic agents. For example, (***S***)-2,3-dihydro-1,4-benzodioxin-2-carboxylic acid is an intermediate for doxazosin mesylate, a primary drug used for the treatment of hypertension and benign prostatic hyperplasia (BPH) [3], 2,3-dihydro-2-hydroxymethyl-1,4-benzodioxin, a prostaglandin D_2_ receptor antagonist [4] and N-[2,4-oxo-1,3-thiazolidin-3-yl]-2,3-dihydro-1,4-benzodioxin-2-carboxamide, which has antihepatotoxic activity [5]. Similarly, (***R***)-2,3-dihydro-1,4-benzodioxin-2-carboxylic acid and its amide is intermediate for WB 4101, an α_1_-adrenoreceptor selective antagonist [6, 7] and MKC242, a potent 5HT_1A_ receptor agonist [8–10]. The absolute configuration of 2,3-dihydro-1,4-benzodioxin-2-carboxylic acid strongly influence the functionality and biological activity of therapeutic agents. For example, the (***S***)-enantiomer of doxazosin is more potent than the (***R***) or racemic for the treatment of BPH [11]. Similarly, (***S***)-enantiomer of WB4101 and derivatives of aminomethylbenzodioxane are stronger antagonists of α_1_-adrenergic receptor compared to the corresponding (***R***)-enantiomer [12–16].

In the literature, preparation of both the enantiomers of 2,3-dihydro-1,4-benzodioxin-2-carboxylic acid has been described by a chemical resolution method using (+)-dehydroabietylamine as chiral base [17]. Not only this method uses a chiral resolving agent, which is rather uncommon and not easily accessible, it suffers from other drawbacks such as low efficiency and non-environment friendly process. An enzymatic kinetic resolution method using esterase from *Serratia marcescens* has also been described for preparation of (***S***)-2,3-dihydro-1,4-benzodioxin-2-carboxylic acid. However, this method leads to less than optimal optical purity, and further enrichment in e.e. by crystallization was not efficient because of unfavourable eutectic point of conglomerate [18]. Therefore, there is a need to develop an efficient method for preparation of enantiomerically pure 2,3-dihydro-1,4-benzodioxin-2-carboxylic acid [19].

**Fig 1:**
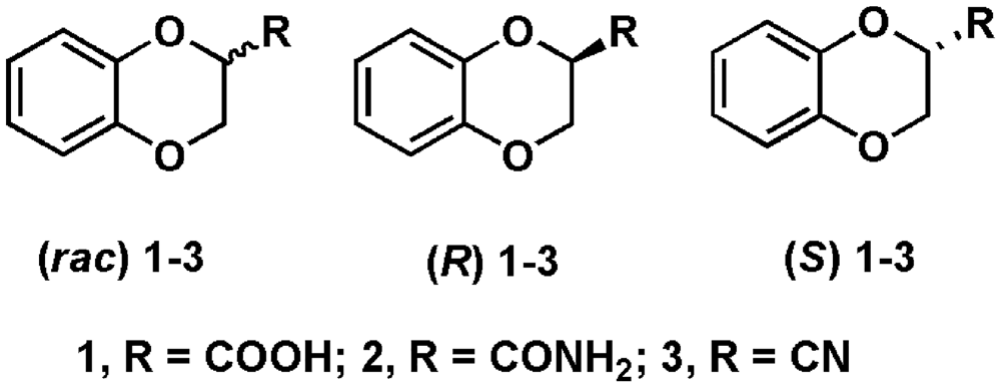
Chemical formulae for compounds 1–3. 2,3-dihydro-1,4-benzodioxin-2-carboxylic acid (**1**), 2,3-dihydro-1,4-benzodioxin-2-carboxamide (**2**) and 2,3-dihydro-1,4-benzodioxin-2-carbonitrile (**3**)

Our interest in finding a biocatalyst for efficient production of enantiomerically pure 2,3-dihydro-1,4-benzodioxane-2-carboxylic acid **(1)** lead us to an amidase activity of *Alcaligenes faecalis* subsp. *parafaecalis*, which produced both ***R*** and ***S*** enantiomers of 2,3-dihydro-1,4-benzodioxin-2-carboxylic acid (**1**) from corresponding racemic amide (**2**) in enantiomeric excess (*e*.*e*.*)* of >99% and with high conversion rate (Fig 1). Following proteomics approach, the amidase of *Alcaligenes faecalis subsp*. *parafaecalis* was identified as indole-3-acetamide hydrolase (IaaH). The gene encoding for IaaH was cloned and expressed in *E*. *coli*. Herein, we describe the findings of this study.

## Materials and Methods

### Materials

All chemicals used in this study were of analytical reagent grade. Ampicillin, isopropyl-β-D-1-thiogalactopyranoside (IPTG), phenyl sepharose, sephacryl S-200 HR, oligonucleotides and other chemicals were purchased from Sigma-Aldrich, Co. (St. Louis, USA). Restriction enzymes, DNA ladders, dNTPs mix and other material used for cloning were obtained from New England Biolabs Inc. (MA, USA). Ni-NTA agarose beads, SDS-PAGE molecular weight marker were purchased from GE Healthcare (UK). DNA gel extraction kit was procured from Qiagen Inc.(CA, USA).

### Bacterial strains and culture conditions

*Alcaligenes faecalis* subsp. *parafaecalis*, a bacterial strain used in this study was isolated in our laboratory from soil sample and deposited with Microbial Type Culture Collection (MTCC), IMTECH, Chandigarh (http://mtcc.imtech.res.in), accession number MTCC 12564. *A*. *faecalis* was routinely grown in rich media comprising tryptone soya broth (TSB, 30 g/L) at 30°C, pH 6.8 for 18 hr at 200 rpm on an orbital shaker. Cells were harvested by centrifugation at 14000×*g* for 15 min at 4°C. The cell pellets were washed thoroughly with 10 mM phosphate buffer, pH 7.2 and re-suspended in the appropriate buffer. For nitrilase activity, 0.1% isobutyronitrile was added to the medium.

*Escherichia coli* (*E*. *coli*) strains were routinely cultured at 37°C, pH 7.0 in Luria-Bertani media (LB, 25 g/L). When needed ampicillin, 100 mg/L was added to media. For induction, isopropyl-β-D-1-thiogalactopyranoside (IPTG) was used at final concentration of 0.2 mM. For the expression of the recombinant protein, *E*. *coli* BL21 (DE3) harbouring plasmid was grown in 10 ml LB media containing ampicillin (100 mg/L) at 37°C, pH 7.0. After 6 hr of growth 1 ml of the culture was inoculated in 100 ml fresh LB media, containing ampicillin and grown at 37°C under 200 rpm shaking condition. When the OD at 600 nm reached 0.6, the culture was induced with 0.2 mM IPTG and incubation continued for another16 hr at 20°C with shaking at 200 rpm on an orbital shaker. The cells were harvested by centrifugation at 14,000×*g* for 15 min.

### Analytical methods

#### Protein Estimation

Bradford’s method was used for protein estimation using bovine serum albumin (BSA) as standard [20]. Bradford’s reagent was utilized according to manufacturer’s instructions. For estimation of protein concentration, samples were diluted in phosphate buffer to a final volume of 100 μL. 100 μL of Bradford’s reagent was added to it. The mixture was maintained at room temperature for 5 min and the absorbance recorded at 595 nm. All assays were carried out in triplicate and appropriate buffer and reagent controls were used for each sample.

#### Assay for amidase activity

Amidase assay was performed in a reaction mixture containing 10 μmol/ml of ***rac***-**2** in 50 mM sodium phosphate buffer, pH 7.0 and an appropriate amount of the enzyme. The reaction was performed at 30°C for 10 min and then stopped by addition of 5N HCl. The contents were extracted in ethyl acetate, dried over anhydrous sodium sulphate and evaporated on rotary evaporator at 45°C under reduced pressure. The sample was then re-dissolved in acetonitrile and analysed for % conversion by HPLC on a system equipped with high pressure gradient dual pump, manual injector, variable temperature column compartment and PDA detector. Column: LiChrospher^®^ RP-18e (Merck Life Science Pvt. Ltd., India), 5μm, 250×4.6 mm; detection UV at 254 nm; elution acetonitrile/water (1:1); temperature 25°C; flow rate 1.0 ml/min. One unit of enzyme activity was defined as the amount of enzyme that catalysed the formation of 1 μmol of ***rac***-**1** per min at 30°C, pH 7.0 from ***rac***-**2**.

#### Determination of enantiomeric excess (e.e.)

Analytical HPLC analyses were performed on a system equipped with high pressure gradient dual pump, manual injector, variable temperature column compartment and PDA detector. E.e. was determined on chiral Column: Chiracel OJ, Daicel, 10 μm, 250×4.6 mm; detection UV at 254 nm; elution hexane/isopropanol/trifluoroacetic acid in ratio of 90:10:0.1; temperature 25°C; flow rate 0.5 ml/min.

#### Colorimetric determination of indole-3-acetamide hydrolase (IaaH) activity

IaaH activity was determined by colorimetric measurement of released ammonia using the Bertholet reaction as described by Vorwerk*et*.*al* [21]. In brief, substrate (5 mM) was incubated with 800 ng of purified protein in a standard buffer, at 30°C in a total volume of 2 ml. After 20 min, aliquots of 100 μl were taken and then 100 μl 0.2 M sodium hypochlorite, 0.33 M sodium phenolate, and 0.1% (w/v) sodium nitroprusside [sodium pentacyanonitrosyl ferrate (III)] were added. The reaction was stopped by heating for 2 min in a boiling water bath, diluted with 600 μl of water and the absorbance was recorded at 640 nm. For the control reaction, heat-denatured enzyme (5 min, 95°C) was used. The amount of released ammonia was determined from the calibration curve prepared from known concentrations of ammonium chloride solutions (0–250 μM).

#### Polyacrylamide gel electrophoresis of proteins

SDS-PAGE was carried out according to the procedure of Laemmli with some modifications [22]. Briefly, the protein samples were prepared by mixing with 1x final concentration of loading sample buffer (0.1 M tris-HCl, pH 6.8, 6% sodium dodecyl sulphate, 30% Glycerol, 15% 2-mercaptoethanol and 0.01% bromophenol blue). Prior to loading to the gel, the samples were heated in a boiling water bath for 5 min. The discontinuous gel system usually had 5% stacking and 12.5% resolving gel. Electrophoresis was carried out using Laemmli buffer at constant current of 15 mA first, till the samples entered into the resolving gel and then at 20 mA till the completion. On completion of electrophoresis, gel was immersed in 0.05% Coomassie Blue R250 in methanol: acetic acid: water (4:1:5) with gentle shaking and was then destained in destaining solution (staining solution without dye) till the background was clear. The protein standards (GE Healthcare, UK) used were phosphorylase b, rabbit muscle (97,000 Da), albumin, bovine serum (66,000 Da), ovalbumin, chicken egg white (45,000 Da), carbonic anhydrase, bovine erythrocyte (30,000 Da), trypsin inhibitor, soybean (20,100 Da), and α lactalbumin, bovine milk (14,400 Da).

#### Native molecular mass of IaaH

The relative molecular mass of the native IaaH was determined by size exclusion chromatography. The Ni-NTA agarose beads purified enzyme (10 μg) was loaded onto a TSKgel G2000SW_XL_ (125 Å, 5μm, 7.8× 300 mm) HPLC column (TOSOH Bioscience LLC, USA). The enzyme was eluted with 100 mM sodium phosphate buffer (pH 8.0) containing 100 mM sodium sulfate at a flow rate of 1 ml/min. The relative molecular mass was then calculated from the calibration curve obtained with standard proteins (MWGF1000 Kit, Sigma-Aldrich, Co., St. Louis, USA).

### Purification of amidase of *Alcaligenes faecalis subsp*. *parafaecalis*

All enzyme purification steps were carried out on fast performance liquid chromatography (FPLC) system (AKTA prime V2.02, GE, Healthcare Pvt Ltd, India) at 4°C.

#### Preparation of cell-free extract

For the preparation of cell-free extract, 10 g cells (wet weight) were suspended in 100 mL of 50 mM phosphate buffer (pH 7.0) containing 1 mM dithiothreitol and disrupted by sonication (22 kHz, in sonic model VCX 750). The cell free extract was obtained by centrifugation at 12,000 rpm for 15 min at 4°C.

#### Ammonium sulphate fractionation

The cell lysate was cooled to 4°C and solid ammonium sulphate was added to 40% saturation. Stirring was continued for another 1 hr and then the precipitate removed by centrifugation at 12000 rpm for 15 min. The supernatant was then brought to 65% saturation with solid ammonium sulphate while maintaining the temperature at 4°C and pH at 7.0. The precipitated proteins were collected by centrifugation at 12000 rpm for 30 min. The pellet was re-dissolved in phosphate buffer, 10 mM pH 7.0 containing 1 M of ammonium sulphate.

#### Phenyl sepharose chromatography

The protein solution from the previous step was applied to a phenyl sepharose 6 fast flow column (15 x 100 mm) pre-equilibrated with 10 mM phosphate buffer (pH 7.0) containing 1 M of ammonium sulphate. The column was washed with the same buffer and eluted with 0.3 M of ammonium sulphate at a flow rate of 1.5 ml/min. Fractions of 5 mL each were collected. The active fractions were pooled and concentrated by ultrafiltration (Millipore-Amicon Ultra15 Centrifugal Filter Units with 10 kDa molecular weight cut-off membrane). The sample was desalted using PD-10 column (GE Healthcare Bio-Sciences Corp, USA).

#### Size exclusion chromatography

0.5 mL of concentrated protein sample from above step was loaded on a sephacryl S200HR gel filtration column (10 x 300 mm, 24 mL bed volume) previously equilibrated with tris-HCl buffer (50 mM, pH 7.0) containing 0.15 M NaCl. The enzyme was eluted with the same buffer at the flow rate of 0.75 mL/min and active fractions were pooled and concentrated by ultracentrifugation using 10 kDa membrane.

### Mass spectrometric analysis of IaaH

A purified protein sample of IaaH was run on 12% SDS-PAGE under reducing conditions. The major band was excised and used for in-gel digestion of protein by trypsin. The Trypsin Profile IGD Kit (Sigma-Aldrich, Co., St. Louis, USA) was used for the preparation of MS/MS sample according to manufacturer’s protocol. These desalted tryptic digested peptides were then mixed with matrix solution consisting of 5 mg/ml of 4-cyanohydroxycinnamic acid (CHCA) in 50% acetonitrile and 0.1% trifluoroacetic acid. MS/MS spectra were acquired using a TOF/TOF^™^ 5800 mass spectrometer (AB Sciex LLC, CA, and USA). Mass spectral data was analysed using the MS/MS ion search of MASCOT program (http://www.matrixscience.com).

### Cloning and expression of IaaH gene from *Alcaligenes faecalis* subsp. *parafaecalis*

The gene sequence encoding for protein IaaH (GenBank accession number: EKU31356.1) was amplified from the genomic DNA of *Alcaligenes faecalis subsp*. *parafaecalis* by PCR using forward IaaHF: 5'ACTGGCTAGCATGTCACTTACCGAACTTAGCG-3' and reverse IaaHR: 5'-AATGCTCGAGTGCCCGTTTGCTCAAAT-3' oligonucleotide pair (underlined bases denote the restriction enzyme site, NheI in forward primer and XhoI in reverse primer). The PCR conditions were, initial denaturation at 95°C for 5 min followed by 30 cycles of 95°C for 60 sec, 58.8°C for 30 sec, 72°C for 1.5 min and final extension at 72°C for 10 min. The PCR product was run on 1.2% agarose gel and intense 1.42 kb band was excised from gel and purified using DNA gel extraction kit. The purified PCR product was digested with NheI and XhoI and run on 1.2% agarose gel and digested fragment excised and again purified using DNA gel extraction kit. Plasmid pET23(a) was digested with NheI and XhoI, and to avoid self-ligation, dephosphorylation was done using calf intestine alkaline phosphatase. T4 DNA ligase was used for ligation of double digested IaaH gene and pET23(a) plasmid and the ligated product was transformed in cloning host *E*. *coli* DH5α. For the expression of protein, the resultant 5 Kb plasmid pET23(a)-IaaH was transformed in expression host *E*. *coli* BL21 (DE3).

### Purification of 6×His-tagged recombinant enzyme

The purification of recombinant IaaH was achieved by affinity chromatography on Ni-NTA agarose beads. All enzyme purification steps were carried out on fast performance liquid chromatography (FPLC) system (AKTA prime V2.02, GE, Healthcare Pvt Ltd, India) at 4°C. *E*. *coli* BL21 (DE3) + pET23(a)-IaaH was cultured as described above. Cells pellet was re-suspended in 25 ml of buffer I (50 mM NaH_2_PO_4_, 300 mM NaCl, 10 mM imidazole and 1 mg/ml lysozyme, pH 8.0) and incubated for 30 min at 4°C. The cell suspension was disrupted by sonication (sonication time, 40 min; 30 s on, 30 s off; ice-cooling). The lysate was centrifuged at 14,000×g for 15 min to remove the cell debris. The cell-free extract was applied onto a Ni-NTA agarose beads packed in column (1.5×10 cm), pre-equilibrated with buffer I. Unbound proteins were washed with buffer II (50 mM NaH_2_PO_4_, 300 mM NaCl and 20 mM imidazole, pH 8.0) and recombinant enzyme eluted with buffer III (50 mM NaH_2_PO_4_, 300 mM NaCl and 100 mM imidazole, pH 8.0). Active fractions were pooled and desalted using Sephadex PD-10 column. This desalted protein sample was concentrated by centrifugation (Amicon Ultra-15 Centrifugal Filter Unit with 30 KDa membrane).

### Experimental procedures

#### General

^1^H NMR spectra were obtained at 300 MHz (Jeol ECX 300) and referenced to TMS (0.0 ppm) or the residual solvent peak (CHCl_3_, 7.26 ppm). Chemical shifts are reported as parts per million (ppm) using the δ scale. ^13^C NMR spectra were recorded at 75 MHz and referenced either to TMS (0.0 ppm) or internal solvent (CDCl_3_, 77.0 ppm). Thin layer chromatography (TLC) was performed on Merck silica gel DC Alurolle Kieselgel 60F_254_ plates and visualized under UV lamp and/or with 0.25% w/v KMnO_4_ and 2% NaHCO_3_ solution in water. Flash column chromatography was carried out using silica gel (200–400 mesh).

#### Preparation of *rac*-2,3-dihydro-1,4-benzodioxin-2-carbonitrile (3)

A two necked 250 ml round bottom flask equipped with a guard tube, magnetic spin bar and dropping funnel was charged with a solution of acrylonitrile (94 mmol) in anhydrous carbon tetrachloride (100 ml). The contents were cooled in ice bath and bromine (94 mmol) added drop wise through the dropping funnel with constant stirring. The mixture was then allowed to warm up to ambient temperature and stirring continued for another 4 hr. The solvents were removed on rotary evaporator under reduced pressure to yield 2,3-dibromopropionitrile in quantitative yield, which was used in next step without further purification.

A solution of 2,3-dibromopropionitrile (10 ml, 100 mmol) in dry acetone (100 ml) was added to a fresh two necked 250 ml round bottom flask equipped with a guard tube and magnetic spin bar and contents heated in oil bath at 55°C. Potassium carbonate (500 mmol) and pyrocatechol (100 mmol) were sequentially added and stirring continued for 3 hr. The reaction was monitored by TLC (ethyl acetate/hexane, 1:4). The solvents were removed under reduced pressure and the contents extracted in ethyl acetate (3×10 ml). The organic layer was washed with 2% sodium hydroxide (5 ml) followed by water (3×5 ml). The organic layer was separated, dried over sodium sulphate and evaporated on rotary evaporator under reduced pressure to leave a residue of ***rac*-3**, which was purified by flash chromatography. Yield 80%; HPLC (column Merck LiChrospher^®^ RP-18e, 5μm, 250×4.6 mm; detection UV at 254 nm; elution acetonitrile/water (1:1); temperature 25°C; flow rate 1.0 ml/min): retention time 5.0 min. ^1^H NMR (300 MHz, CDCl_3_): δ = 6.95 (m, 4H), 5.12 (m, 1H), 4.38 (m, 1H). ^13^C NMR (75 MHz, CDCl_3_): δ = 142.37, 140.59, 123.59, 122.74, 117.87, 114.84, 64.78, 61.99.

#### Preparation of *rac*-2,3-dihydro-1,4-benzodioxin-2-carboxamide (2)

A solution of *rac*-**3** (0.1mol) in DMSO (30 ml) was cooled in an ice bath. To the stirred solution, anhydrous K_2_CO_3_ (2 g) followed by 30% H_2_O_2_ (12 ml) were added. The mixture was then allowed to warm up to ambient temperature (25°C) and stirring continued for another 15 min. Distilled water (50 ml) was added and contents stirred for another 20 min. The progress of the reaction was monitored by TLC (ethyl acetate/hexane, 1:1). After the completion of the reaction, the contents were extracted with ethyl acetate (2x25 ml), organic layer separated and washed with water (3×5 ml), dried over sodium sulphate and evaporated on rotary evaporator under reduced pressure to yield ***rac*-2**, which was purified by flash chromatography [23]. Yield 90%; HPLC (column Merck LiChrospher^®^ RP-18e, 5μm, 250×4.6 mm; detection UV at 254 nm; elution acetonitrile/water (1:1); temperature 25°C; flow rate 1.0 ml/min): retention time 3.7 min. ^1^H NMR (300 MHz, CDCl_3_): δ = 6.93 (m, 4H), 6.47 and 6.26 (each bs, each 1H), 4.68 (dd, *J* = 7.2, 2.7 Hz, each 1H), 4.94 (dd, *J* = 11.4, 2.7 Hz, 1H), 4.23 (dd, *J* = 11.4, 7.2 Hz, 1H). ^13^C NMR (75 MHz, CDCl_3_): δ = 170.35, 143.32, 141.69, 122.55, 122.10, 117.80, 117.20, 73.23, 65.16.

#### *A*. *faecalis* subsp. *parafaecalis* catalysed preparation of (*S*) and (*R*)-2,3-dihydro-1,4-benzodioxin-2-carboxylic acid (1) and (*S*)-2,3-dihydro-1,4-benzodioxin-2-carboxamide (2)

1.2 gm cells (wet weight) of *A*. *faecalis* subsp. *Parafaecalis* MTCC 12564 were suspended in 5 ml 50 mM bicarbonate buffer (pH 11.4). *rac*-**2** (0.31 mmol) was added to the cell suspension and contents incubated at 30°C on an orbit shaker at 200 rpm for 10 min. The contents were then extracted in ethyl acetate (2x5 ml). The ethyl acetate layer was then extracted with cold 2% NaOH (2x5 ml). The organic layer was washed with cold water (5 ml), dried over anhydrous sodium sulphate and evaporated under reduced pressure on a rotary evaporator to yield (***S***)-2,3-dihydro-1,4-benzodioxin-2-carboxamide (**2**), which was characterized by ^1^H NMR (data same as that reported for ***rac*-2** above).

The aqueous layer was acidified with 2N HCl to pH 2.0 in cold and re-extracted in ethyl acetate (2x5 ml). The organic layer was washed with cold water (5 ml), dried over anhydrous sodium sulfate and evaporated under reduced pressure on a rotary evaporator to yield (***R***)-2,3-dihydro-1,4-benzodioxin-2-carboxylic acid (**1**). ^1^H NMR (300 MHz, CDCl_3_): δ = 6.99 (m, 1H), 6.88 (m, 3H), 4.85 (m, 1H), 4.40 (m, 1H). ^13^C NMR (75 MHz, CDCl_3_): δ = 170.72, 143.09, 142.42, 122.15, 121.81, 117.43, 71.82, 64.96. (***S***)-2,3-dihydro-1,4-benzodioxin-2-carboxamide (**2**) was subjected to biocatalysed hydrolysis with *A*. *faecalis* subsp. *parafaecalis* in bicarbonate buffer, pH 11.4 at 30°C. The contents were then acidified to pH 2.0 with 2N HCl and then extract with ethyl acetate. The organic layer was washed with cold water (5 ml), dried over anhydrous sodium sulfate and evaporated under reduced pressure on a rotary evaporator to yield (***S***)-2,3-dihydro-1,4-benzodioxin-2-carboxylic acid (**1**), which was characterized by ^1^H NMR (data same as that reported for (***R***)-2,3-dihydro-1,4-benzodioxin-2-carboxylic acid above).

## Results and Discussion

Recently, our group has described a nitrilase activity from *Alcaligenes faecalis* subsp. *parafaecalis* that caused dynamic kinetic hydrolytic resolution of mandelonitrile to produce (***R***)-mandelic acid in 100% e.e. at 100% conversion rate [24]. Initially, we explored this nitrilase for preparation of enantiopure 2,3-dihydro-1,4-benzodioxin-2-carboxylic acid (**1**) from corresponding *rac*-nitrile (**3)**. Accordingly, *rac*-**3** (0.31mmol) was incubated at 30°C with 1.2 gm cells (wet weight) of *A*. *faecalis* subsp. *parafaecalis* in 5 ml 50 mM phosphate buffer (pH 7.0) with shaking at 200 rpm on an orbital shaker. The progress of reaction was monitored by TLC. The enzyme proved to be a very efficient hydrolase for this substrate, converting 100% substrate to the product in 4 hr. Enantiomeric excess was determined by chiral HPLC as described in materials and methods section. However, we were disappointed to note that in sharp contrast to mandelic acid that was produced in 100% e.e., the e.e. of 2,3-dihydro-1,4-benzodioxin-2-carboxylic acid (**1**) was 0%.

**Fig 2:**
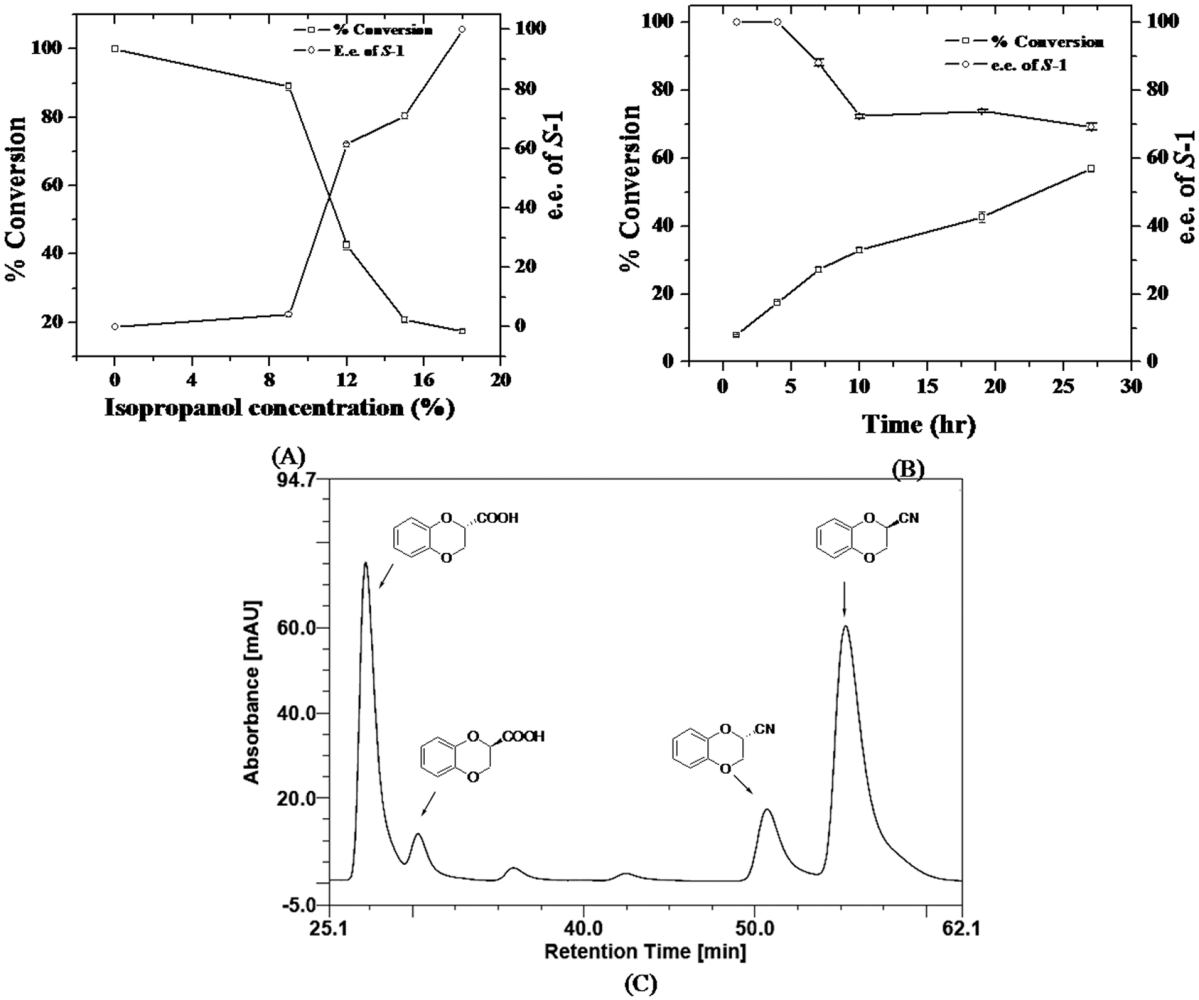
Effect of isopropanol on activity and enantioselectivity of *A*. *faecalis* nitrilase. (A) The effect of isopropanol concentration (0–18% v/v) on e.e and % conversion. The reaction was done in phosphate buffer, pH 7.0 at 30°C in presence of varying amounts of isopropanol. (B) Conversion rates and e.e. in presence of isopropanol 18% (v/v) at various time points (0 to 27 hr). (C) HPLC trace of reaction in presence of isopropanol 18% (v/v) at 50% conversion. Column: Chiracel OJ, 10 μm, 250×4.6 mm; detection UV at 254 nm; elution hexane/isopropanol/trifluoroacetic acid in ratio of 90:10:0.1; temperature 25°C; flow rate 0.5 ml/min.

It is known in literature that additives such as alcohols can dramatically alter enantioselectivity of an enzyme [25, 26]. Therefore, we attempted the nitrilase catalysed conversion of *rac*-**3** to **1** under the above reaction conditions but in presence of varying concentrations (0 to 18% v/v) of isopropanol (IPA). The results are summarized in Fig 2A. There was no significant improvement in e.e. at IPA concentration of up to 9%. Increasing the concentration of IPA from 9 to 18% resulted in gradual increase in e.e., but with concomitant decrease in conversion rate. The e.e was about 60 and 70%, respectively at IPA concentration of 12 and 15%. The corresponding conversion rate was 42 and 22%, respectively. At IPA concentration of 18%, the e.e improved to the desired 100%, but the conversion rate was only 18%. Attempts to optimize conversion rate while achieving high e.e. by increasing the reaction time proved futile. Increasing the reaction time resulted in improved conversion, but with simultaneous decrease in e.e. of the product. The best result was about 70% e.e. at 50% conversion in 27 h (Fig 2B and 2C). The absolute configuration was assigned as ***S*** to major enantiomer based on comparison of retention times with literature [19]. Thus, use of IPA as additive in biocatalyzed reaction proved useful in improving e.e., but was not optimal for production of enantiopure **1**.

**Fig 3:**
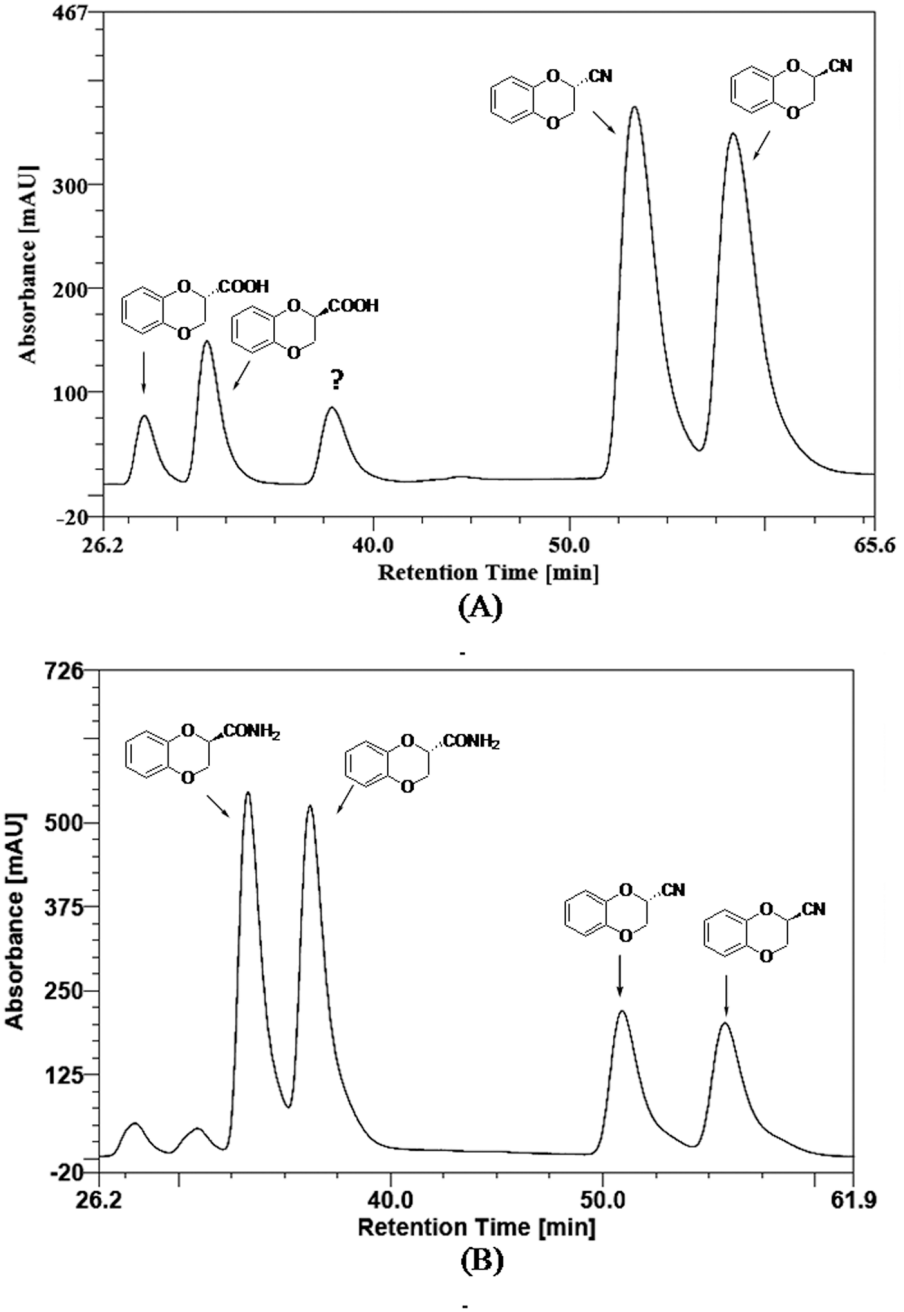
Effect of cyclohexyl amine (CHA) on activity and e.e. of *A*. *faecalis* nitrilase. (A) Chiral HPLC trace (Chiracel OJ, 10 μm, 250×4.6 mm; detection UV at 254 nm; elution hexane/isopropanol/trifluoroacetic acid in ratio of 90:10:0.1; temperature 25°C; flow rate 0.5 ml/min) showing inversion in configuration and appearance of unidentified peak (marked?) in reaction of *rac*-nitrile **3** in phosphate buffer, pH 7.0 at 30°C in presence of 3% CHA. (B) Chiral HPLC trace of reaction done under similar conditions as in A, but in absence of biocatalyst (control reaction).

Next, we attempted use of cyclohexylamine (CHA) to improve e.e. and productivity via dynamic kinetic resolution [27, 28]. While monitoring the reaction by HPLC at CHA concentration of 3%, we noticed that at about 25% conversion, the product acid was obtained in about 70% e.e., but the major enantiomer had ***R*** configuration as opposed to IPA addition which produced major enantiomer in ***S*** configuration (Fig 3A). At 3% CHA, the pH of reaction was found to be 9.7. This interesting observation, i.e. inversion in enantioselectivity of enzyme with CHA prompted us to have a closer look at the results. The HPLC clearly showed presence of an intermediate, which was the main product in control reaction run under similar conditions but without biocatalyst (Fig 3B). Since the pH of the reaction reached 9.7 in presence of 3% CHA, base catalysed hydration of nitrile to amide is quite feasible. The structure of the intermediate was confirmed as amide by comparison of its NMR and HPLC data with authentic sample of amide prepared as described in materials and methods section [23]. Another observation worth noticing was that the amide produced in biocatalyzed reaction eluted as single peak (100% e.e.) compared to the control in which it eluted as two peaks in roughly equal ratio (racemic). These results can possibly be rationalized by assuming that the acid (**1**) is produced by two routes, (i) by action of nitrilase with poor ***S***-selectivity and (ii) action of an amidase with high ***R***-selectivity.

**Fig 4:**
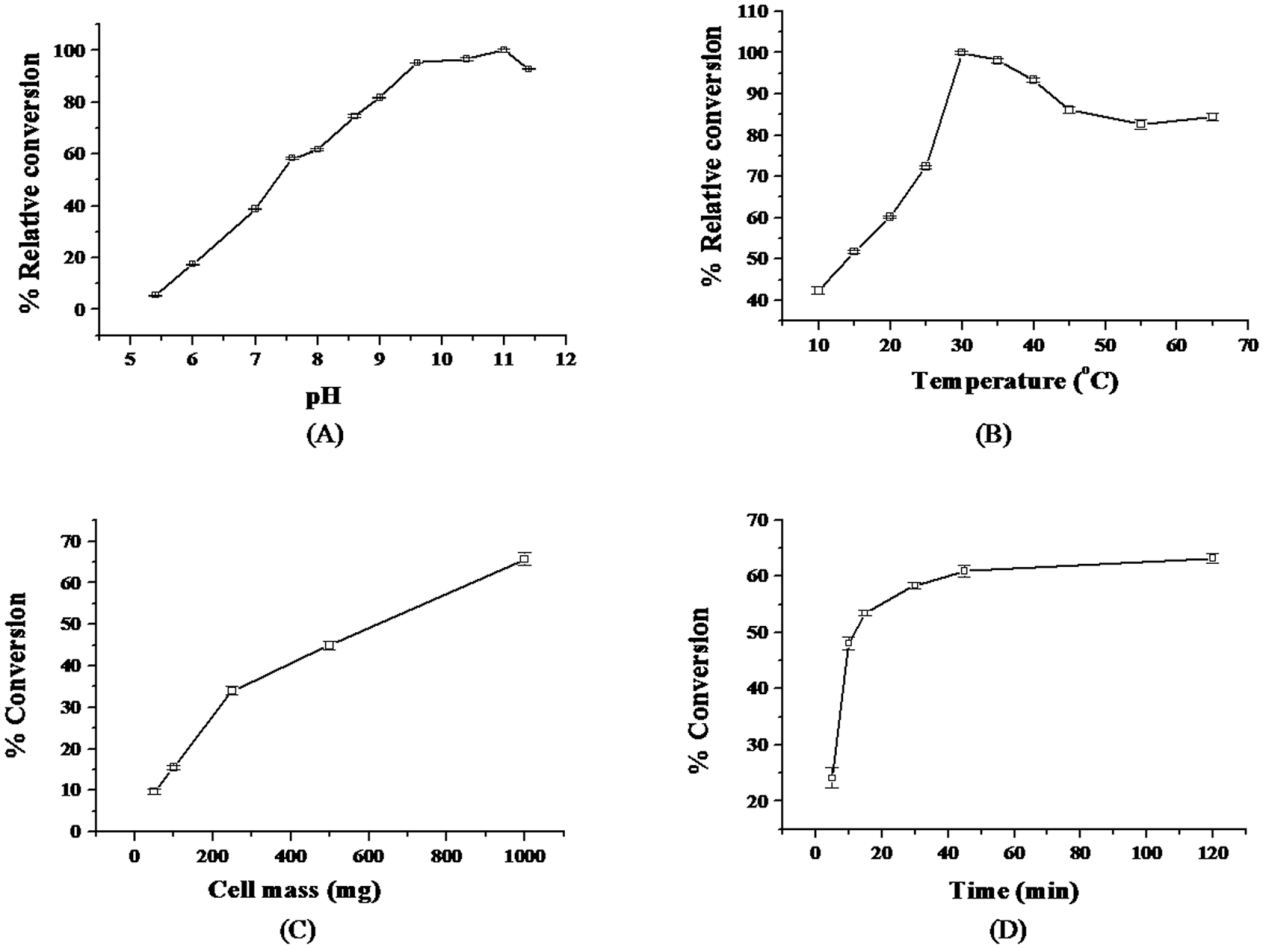
Effect of reaction conditions on *A*. *faecalis* catalysed conversion of *rac*-amide 2. Effect of pH (A), temperature (B) and cell mass (wet cell basis) (C) on *A*. *faecalis* catalysed conversion of *rac*-amide **2**. (D) Conversion of *rac*-amide **2** in 50 mM bicarbonate buffer, pH 11.4 at 30°C at various time points (0 to 12 hr).

This chance discovery, lead us to follow the amidase route for preparation of enantiopure **1**. Biocatalyzed reaction was performed as described above using ***rac*-2** as substrate in place of ***rac***-**3**. The reaction was done in phosphate buffer, pH 7.0 at 30°C using 1.0 g cells (wet cell mass). The progress of the reaction was monitored by TLC and e.e. was determined by chiral HPLC. A rapid conversion of amide to acid occurred till about 50% conversion in 10 min, after which the reaction became sluggish. These results confirmed the presence of an amidase activity in *Alcaligenes faecalis* subsp. *parafaecalis*. Optimal reactions conditions for enzymatic hydrolysis were established by performing the reaction under various conditions of pH, temperature, cell mass and time period. The buffers used were 50 mM citrate (pH 4.0 to 6.0), 50 mM sodium phosphate (pH 6.0 to 8.0), 50 mM tris-HCl (pH 7.0 to 9.0), 50 mM glycine-sodium hydroxide (pH 8.5 to 10.0) and 50 mM sodium bicarbonate pH (10.5 to 11.9). Although the enzyme had maximum activity at pH 11.0, it retained about 90% of maximal activity in pH range of 9.5 and 11.4 at 30°C (Fig 4A). The activity was 70–80% in the pH range of 8.0 and 9.0. The optimal temperature was 30°C, but the enzyme retained about 90% activity at temperatures up to 65°C (Fig 4B). The conversion rates were proportional to the cell mass (Fig 4C). The initial rate of reaction was high, reaching about 50% conversion in 10 min at pH 11.4 at 30°C (wet cell mass 1.2 g), after which the reaction slowed down considerably, requiring 120 min to reach 60% conversion (Fig 4D).

**Fig 5:**
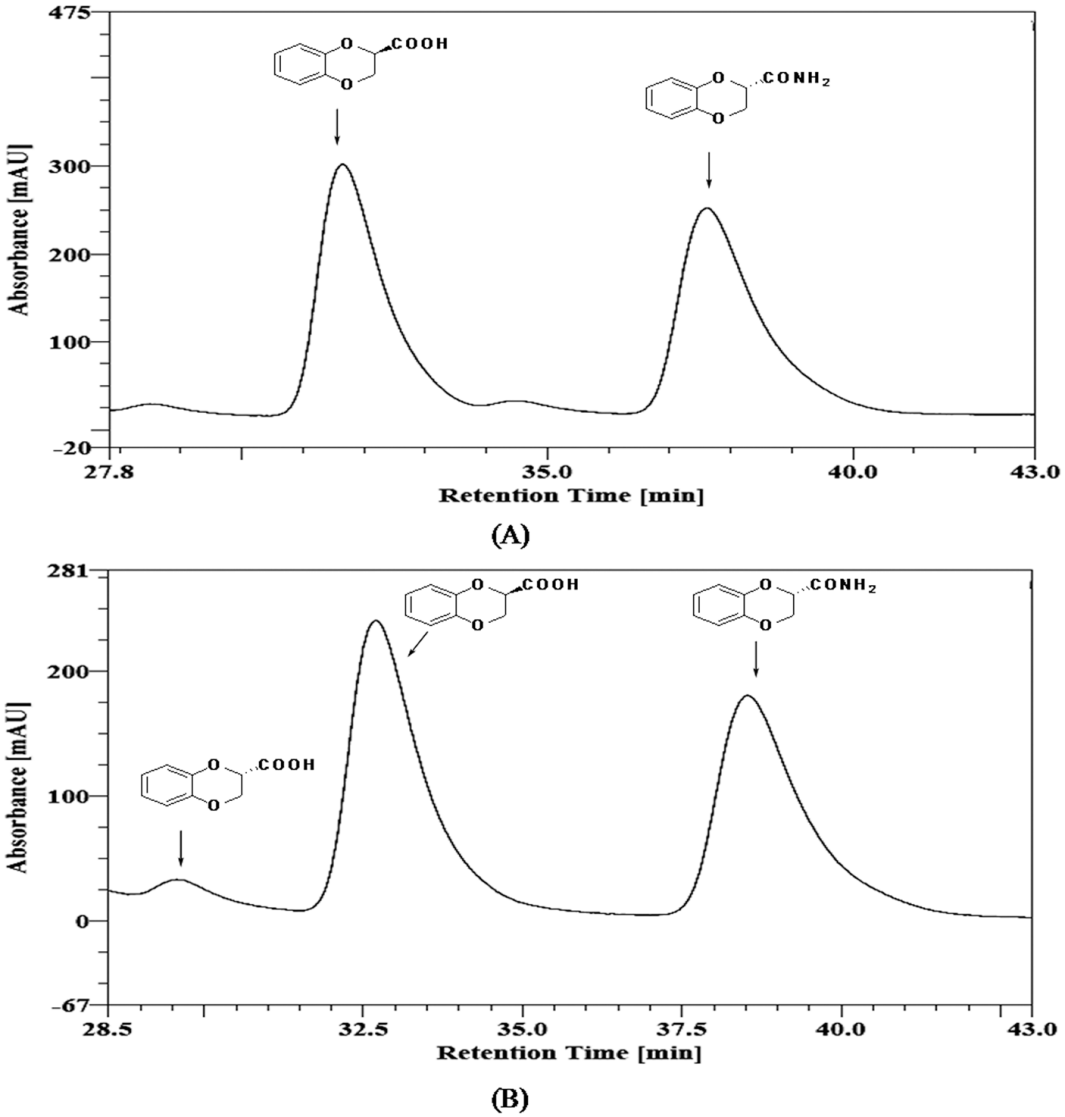
*A*. *faecalis* catalysed asymmetric resolution of *rac*-2. Chiral HPLC trace (Chiracel OJ, 10 μm, 250×4.6 mm; detection UV at 254 nm; elution hexane/isopropanol/trifluoroacetic acid in ratio of 90:10:0.1; temperature 25°C; flow rate 0.5 ml/min) of reaction of ***rac***-**2** in sodium carbonate buffer, pH 11.4 at 30°C at ~50% conversion (A) and 53% conversion (B).

For practical applications, it was considered advantageous to use pH 11.4 for the reaction, since the product carboxylic acid is expected to remain soluble under these conditions and the expected drop in pH during the progress of reaction would not significantly affect the rate of reaction at pH of up to 8.0. Thus, *rac*-amide **2** (0.31 mmol) was incubated with a suspension of 1.2 gm cells (wet cell weight) in 5 ml 50 mM bicarbonate buffer (pH 11.4) at 30°C with shaking at 200 rpm on an orbital shaker. The progress of the reaction was monitored by HPLC. The reaction was stopped at about 50% conversion (10 min). The e.e. of the acid and remaining amide was determined to be >99% (configuration ***R***) and 99% (configuration ***S***), respectively (Fig 5A). To obtain ***S***-amide in 100% e.e., the reaction was allowed to proceed to about 53% conversion (Fig 5B). These results indicated that the amidase is highly selective for ***R***-amide, however the selectivity was not absolute. The ***S***-amide was hydrolysed at much slower rate. For irreversible reactions, such as amide hydrolysis in aqueous medium, the enantioselectivity of an enzyme for kinetic resolution under a given set of conditions can be defined in terms of a dimensionless parameter, Enantiomeric Ratio (*E*) [29]. An *E* value of >30 is considered excellent for enantioselective enzymatic resolution. *E* value for *A*. *faecalis* catalysed hydrolysis of *rac*-**2** under these conditions was calculated using the computer program developed by Faber *et*. *al*. and was found to be >200 [30].

The separation of ***R***-acid and ***S***-amide was achieved by simple two phase partition between 2% sodium hydroxide and ethyl acetate. ***S***-amide was recovered from ethyl acetate phase. The ***R***-acid was recovered from alkali phase by cooling it to 4°C followed by addition of 2N hydrochloric acid till pH reached 2.0. At 50% conversion, amide (***S***)-**2** and acid (***R***)-**1** were isolated in 47.3 (e.e. 99%) and 48.8% (e.e. >99%) yield, respectively. However, when the reaction was stopped at 53% conversion, (***S***)-**2** and (***R***)-**1** were obtained in 46.2 (e.e. 100%) and 50.6 (e.e. 94%) yield, respectively. The products, (***S***)-**2** and (***R***)-**1** were characterized by NMR. Amide protons in (***S***)-**2** appeared as two distinct broad singlets at δ 6.47 and 6.26 in ^1^H NMR. These signal were absent in ^1^H NMR of (***R***)-**1**. Single H2 proton and two H3 protons in (***S***)-**2** resonated at δ 4.68 (dd, *J* = 7.2 and 2.7 Hz), 4.49 (dd, *J* = 11.4 and 2.7 Hz) and 4.23 (dd, *J* = 11.4 and 7.2 Hz), whereas in (***R***)-**1**, these resonated as multiplets at 4.85 and 4.40. The aromatic protons in (***S***)-**2** appeared as single multiplet at δ 6.93, whereas in (***R***)-**1**, these appeared as two multiplets at δ 6.99 and 6.88. The recovered ***S***-amide of 100% e.e., when subjected to biocatalyzed hydrolysis using the same biocatalyst, but for longer period of time (20 hr) resulted in complete conversion to ***S***-acid without loss of e.e. Thus, depending on reaction conditions, both the enantiomers of 2,3-dihydro-1,4-benzodioxin-2-carboxylic acid (**1**) are accessible in >99% e.e.

### Isolation of the amidase activity and its characterization as indole-3-acetamide hydrolase (IaaH)

**Fig 6:**
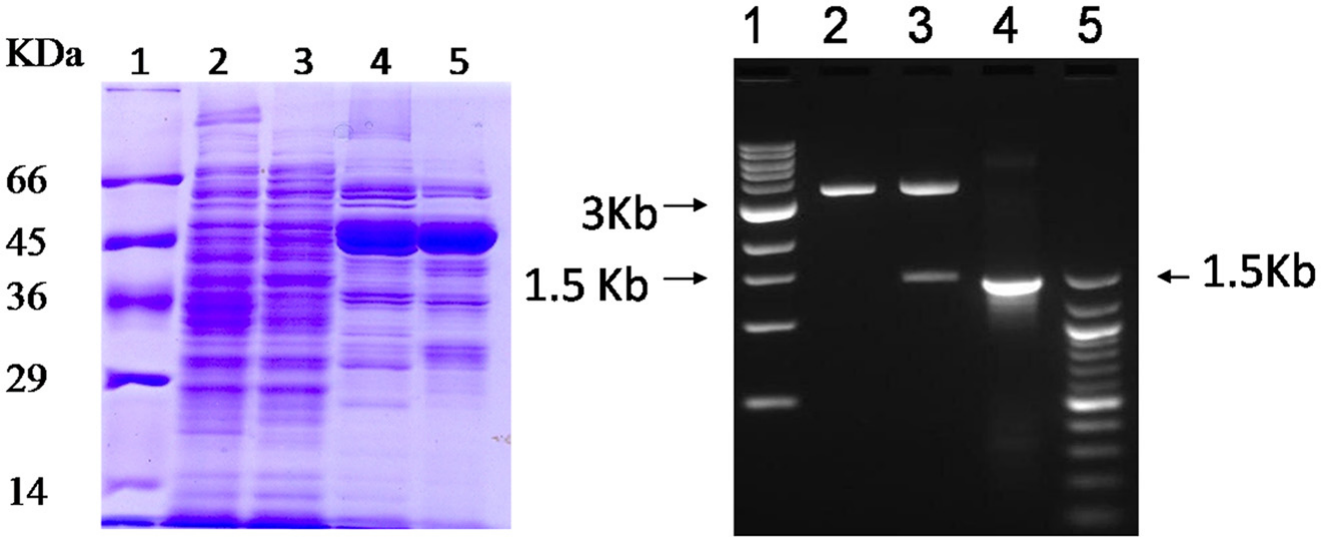
SDS-PAGE of IaaH at various purification steps and cloning and expression of *IaaH* in *E*. *coli*. (A) Protein fractions were run under reducing conditions on 12% SDS-PAGE. Lane 1, molecular weight markers; lane 2, cell-free extract; lane 3, ammonium sulphate fractionation (40–65%); lane 4, phenyl sepharose chromatography; lane 5, size exclusion chromatography. (**B)** Electrophoresis was done on 1.2% agarose gel. Lane 1, 1 Kb ladder; lane 2, Nhe1-Xho1 digested pET23(a); lane 3, Nhe1-Xho1 digested 1.42 Kb IaaH in pET23(a); lane 4, Nhe1-Xho1 digested 1.42 Kb PCR amplified *IaaH*; lane 5, 100 bp ladder.

The isolation and purification of amidase from *Alcaligenes faecalis* subsp. *parafaecalis* was attempted as described in materials and methods section. Briefly, it involved ammonium sulphate fractionation (40–65% saturation) followed by hydrophobic interaction chromatography using phenyl sepharose 6 fast flow column (Sigma-Aldrich, Co., St. Louis, USA) and gel permeation chromatography on sephacryl S200HR gel filtration column (Sigma-Aldrich, Co., St. Louis, USA). The results are summarized in Table 1. The enzyme activity was determined as described in materials and methods section using ***rac***-**2** as substrate. One unit of enzyme activity was defined as the amount of enzyme that catalysed the formation of 1 μmol min^-1^ of ***rac***-**1** at 30°C, pH 7.0 from ***rac***-**2**. Overall a 13.2-fold purification was achieved with a recovery of 10.3%. The specific activity of purified enzyme preparation was 4U mg^-1^ protein at pH 7.0 and 30°C. SDS-PAGE of the protein run under reducing conditions showed that enzyme was only partially purified (Fig 6A).

**Table 1:** Summary of purification steps of the IaaH from *Alcaligenes faecalis subsp*. *parafaecalis*

Purification step	Total Protein (mg)	Total Activity μmolmin^-1^	Specific activity μmolmin^-1^ mg^-1^	Yield (%)	Purificatio (fold)
Cell-free extract	180	54	0.301	100	1
Ammonium sulphate fractionation (40–65%)	63	30.24	0.48	55	1.6
Hydrophobic interaction chromatography	3.5	12.9	3.69	23.8	12.3
Gel filtration chromatography	1.4	5.6	4	10.3	13.2

The prominent band from SDS-PAGE run under reducing conditions was excised and subjected to in-gel digestion with trypsin. Mass spectral data of a sample of desalted tryptic digest was obtained as described in materials and methods section and analysed using the MS/MS ion search of MASCOT program (http://www.matrixscience.com). Out of the top 10 protein matches in the outcome, only one was found to be an amidase enzyme, which has been described as indole acetamide hydrolase (IaaH) from *Alcaligenes sp*. HPC1271 (GenBank accession number: EKU31356.1). We performed *in-silico* trypsinization of indole acetamide hydrolase of *Alcaligenes sp*. HPC1271 (http://web.expasy.org/peptide_mass/) and compared the data with that of trypsin digest of the amidase of *Alcaligenes faecalis* subsp. *parafaecalis*. Three peptide sequences of tryptic digest of amidase showed excellent match with *in-silico* tryptic digest of IaaH, suggesting that the amidase of this study may be IaaH. The sequences were YSGQGIVPLSPTR (*Mr* = 1374.6029), QFPAGLGTDTGASVR (*Mr* = 1476.6035) and SVMHELAFGITTNNATTGPSR (*Mr* = 2203.8774).

### Molecular cloning of *IaaH* gene from *Alcaligenes faecalis* subsp. *parafaecalis* and its functional expression in *E*. *coli* BL21 (DE3)

Draft genome sequence of *Alcaligenes sp*. HPC1271 was accessible in NCBI database (http://www.ncbi.nlm.nih.gov). The gene sequence encoding for indole acetamide hydrolase is available in the database (GenBank accession number: EKU31356.1), based on which we designed primers (IaaHF and IaaHR) for cloning of amidase of *Alcaligenes faecalis* subsp. *parafaecalis*. The IaaH gene was amplified using genomic DNA of *Alcaligenes faecalis* subsp. *parafaecalis* as template and oligonucleotide pair IaaHF and IaaHR as primers in polymerase chain reaction (PCR) using. The amplified DNA fragment was digested with NheI and XhoI and then ligated into double digested (NheI and XhoI) pET23(a) vector by T4 DNA ligase. The ligated product was transformed into competent *E*. *coli* DH5α. For the confirmation of cloning of IaaH gene, plasmid was isolated from *E*. *coli* DH5α grown on LB broth containing ampicillin. The plasmid was then double digested with NheI and XhoI to yield 3.6 Kb pET23(a) backbone and 1.42 Kb insert (Fig 6B), which suggested that *IaaH* gene was cloned downstream of *lac* promoter of pET23(a). DNA sequencing of the recombinant plasmid was done to confirm this result. For the expression of IaaH, pET23(a)-IaaH was transformed into expression host *E*. *coli* BL21 (DE3) resulting in *E*. *coli* BL21 (DE3)+pET23(a)-IaaH. *E*. *coli* BL21 (DE3)+pET23(a)-IaaH was grown and cell-free extract obtained as described in materials and methods section. The cell-free extract catalysed the conversion of ***rac***-**2** to ***R***-**1** under the reaction conditions described above for whole-cell biocatalysed reaction with *Alcaligenes faecalis* subsp. *parafaecalis*. These results confirmed the identity of amidase of *Alcaligenes faecalis* subsp. *parafaecalis* as IaaH.

### Purification and characterization of recombinant IaaH

**Fig 7:**
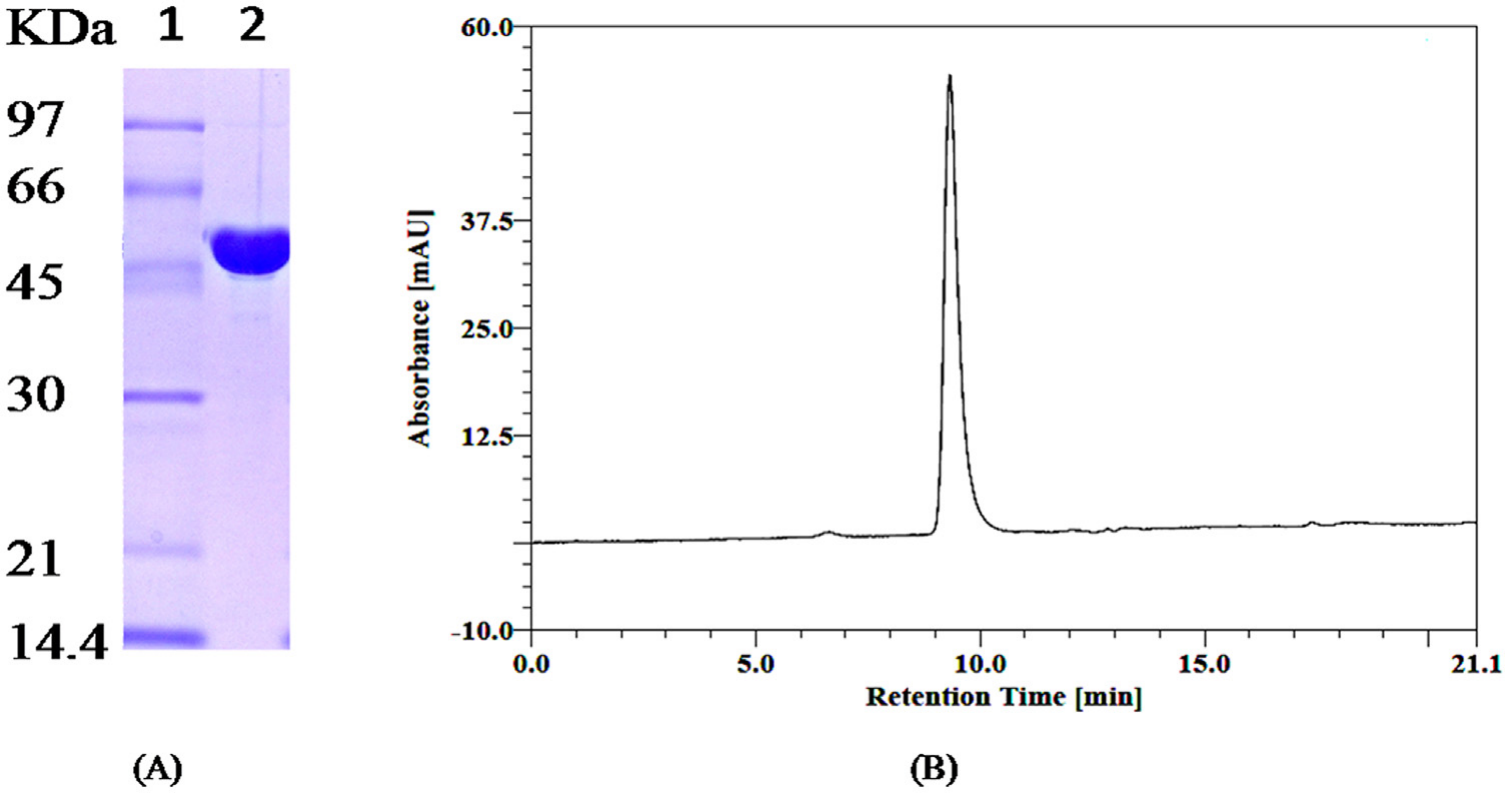
Molecular mass of recombinant IaaH. (A) SDS PAGE of IaaH was run under reducing conditions on 12% SDS-PAGE. Lane 1, MW Marker and Lane 2, Ni-NTA agarose beads purified IaaH. (B) Elution profile of purified enzyme on TSKgel G2000SW_xL_ column.

As a result of the N-terminal 6× His affinity tag, the recombinant enzyme was simply purified by affinity chromatography using a Ni-NTA agarose beads. The purified enzyme was found to be electrophoretically homogeneous and exhibited a single band when run on SDS-PAGE under reducing conditions with a molecular weight of ~49 KDa (Fig 7A). The molecular mass of native IaaH was determined to be ~50 KDa (Fig 7B) by gel filtration chromatography using TSKgel G2000SW_XL_ (125 Å, 5μm, 7.8× 300 mm) HPLC column (TOSOH Bioscience LLC, USA). This result suggested that the IaaH exist in a monomeric form similar to its homolog amidase 1 in *Arabidopsis thaliana* [31, 32].

**Fig 8:**
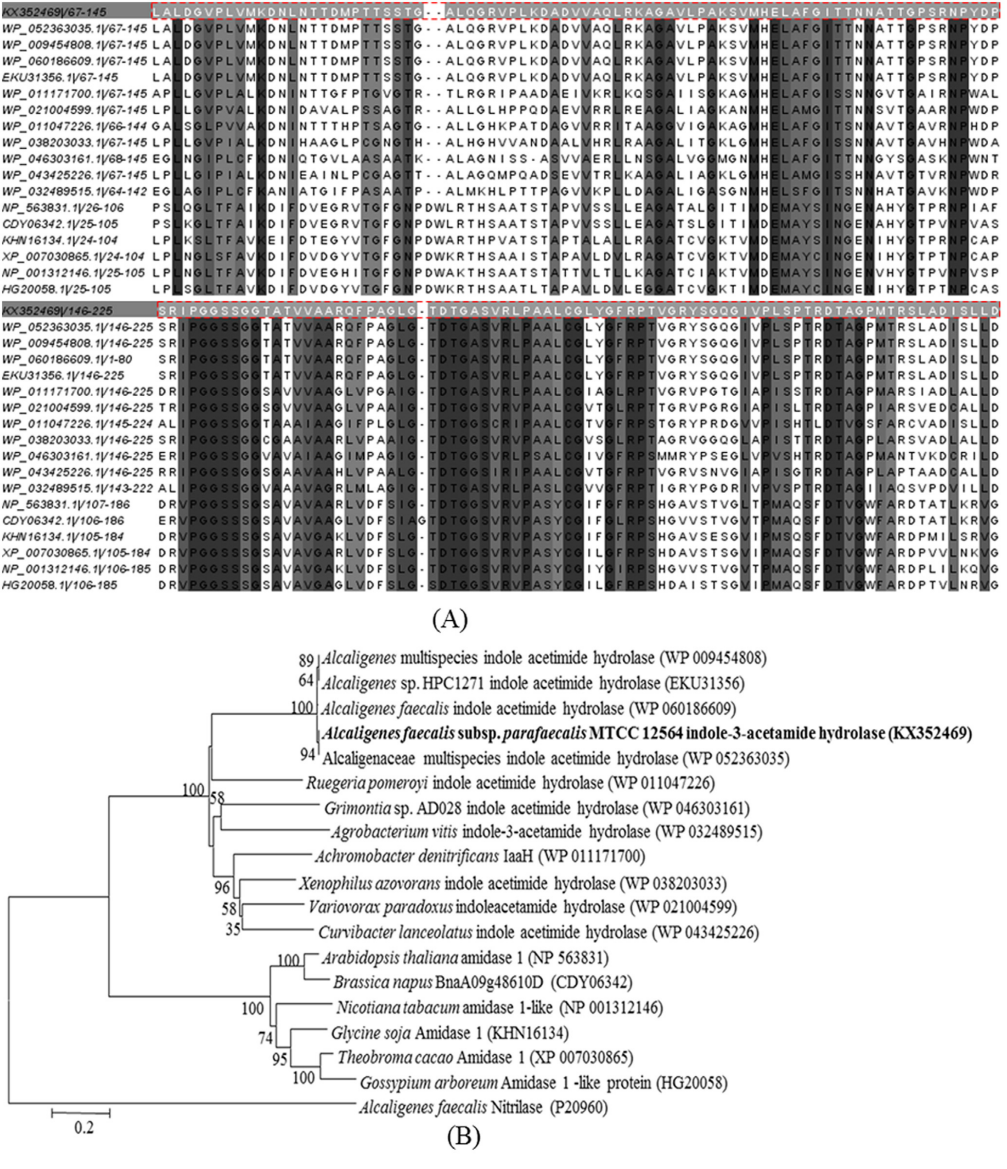
Homology and evolutionary analysis of IaaH of *Alcaligenes faecalis* subsp. *parafaecalis* MTCC 12564. (A) Amino acid sequence alignment of IaaH of this study (GenBank accession number: KX352469, dotted box) with other indole-3-acetamide hydrolases of bacterial and plant origin around the amidase signature motif. The alignment was generated with CLUSTALW and viewed with Jalview. Identical residues present in all aligned sequences are marked in black and identical residues present in more than 50% of the aligned sequences are marked in grey. (B) Phylogenetic analysis of IaaH of this study (in bold letters) with other indole acetamide hydrolases of bacterial and plants. Phylogenetic tree was constructed using neighbour-joining method with CLUSTALW in MEGA 6.0. Bootstrap values were calculated by taking 1000 replicates are shown next to the branches. 0.2 substitution per amino acid position is represented by bar. Nitrilase from *Alcaligenes faecalis* was taken as an out-group. The NCBI accession numbers of enzymes are represented in parentheses.

The amino acid composition and sequence analysis exhibited that IaaH has conserved stretch of ~140 amino acids, rich in glycine and serine, which is characteristic of amidase sequence (AS) family members [33]. The involvement of Ser-*cis*Ser-Lys catalytic triad in the hydrolysis of amide bond in AS family of enzymes has been established based on crystal structure analysis and site directed mutagenesis studies done on various members of this class of enzymes [34–37]. To examine the homology of the enzyme with other indole-3-acetamide hydrolases from bacteria and plant species, the amino acid sequence of IaaH of *Alcaligenes faecalis* subsp. *parafaecalis* (GenBank accession number: KX352469) was used for BLAST search in the non-redundant protein sequence (nr) NCBI database. Eleven sequences that showed highest identity with IaaH of *A*. *faecalis* subsp. *parafaecalis* were selected (Table 2). However, this search did not return any match from sequences of plant origin. In order to obtain sequences from plants for comparison, BLAST search was done using amino acid sequence of amidase 1 from *Arabidopsis thaliana* (homologue of IaaH) and six sequences showing highest identity score were selected (Table 2). CLUSTALW analysis for Multiple Sequence Alignment (MSA) of all these 17 sequences suggested that all the sequences have the common amidase signature motif along with catalytic triad (Fig 8A). In phylogenetic analysis, amino acid sequences can be easily distinguishable in two parts based on their origin in either bacteria or plant, thus establishing the evolutionary relationship of enzyme with other homologs in bacteria and plants (Fig 8B). Sequence of IaaH of this study (GenBank accession number: KX352469) was 99% identical with the sequence of IaaH of *Alcaligenes sp*. HPC1271 (GenBank accession number: EKU31356.1). IaaH of this study was amplified based on the sequence of IaaH of *Alcaligenes sp*. HPC1271.

**Table 2:** *A*. *faecalis* IaaH homologs in bacteria and plants.

Source organism	Accession no.	Number of deduced amino acid	% Identity*	E-value
Plant AMI1 proteins				
*Glycine soja*	KHN16134.1	433	44	8e-^30^
*Theobroma cacao*	XP_007030865.1	432	44	3e-^27^
*Nicotiana tabacum*	NP_001312146.1	425	40	9e-^30^
*Arabidopsis thaliana*	NP_563831.1	425	29	3e-^31^
*Brassica napus*	CDY06342.1	422	27	3e-^27^
*Gossypium arboreum*	KHG20058.1	432	26	4e-^27^
Bacterial indole acetamide hydrolase				
*Alcaligenaceae* Multispecies	WP_052363035.1	472	100	0
*Alcaligenes faecalis*	WP_060186609.1	472	99	0
*Alcaligenes* Multispecies	WP_009454808.1	472	99	0
*Alcaligenes* sp. HPC1271	EKU31356.1	472	99	0
*Ruegeria pomeroyi*	WP_011047226.1	459	47	3e-^131^
*Achromobacter denitrificans*	WP_011171700.1	473	46	4e^-135^
*Xenophilus azovorans*	WP_038203033.1	467	46	3e^-126^
*Variovorax paradoxus*	WP_021004599.1	468	46	1e-^120^
*Curvibacter lanceolatus*	WP_043425226.1	468	44	5e^-121^
*Grimontia* sp. AD028	WP_046303161.1	468	42	1e^-122^
*Agrobacterium vitis*	WP_032489515.1	472	42	1e-^120^

*% identity were determined by aligning the sequence of IaaH of this study (GenBank accession number: KX352469) with the proteins listed in Table 2.

### Substrate spectrum for indole-3-acetamide hydrolase (IaaH) of *Alcaligenes faecalis* subsp. *parafaecalis*

**Fig 9:**
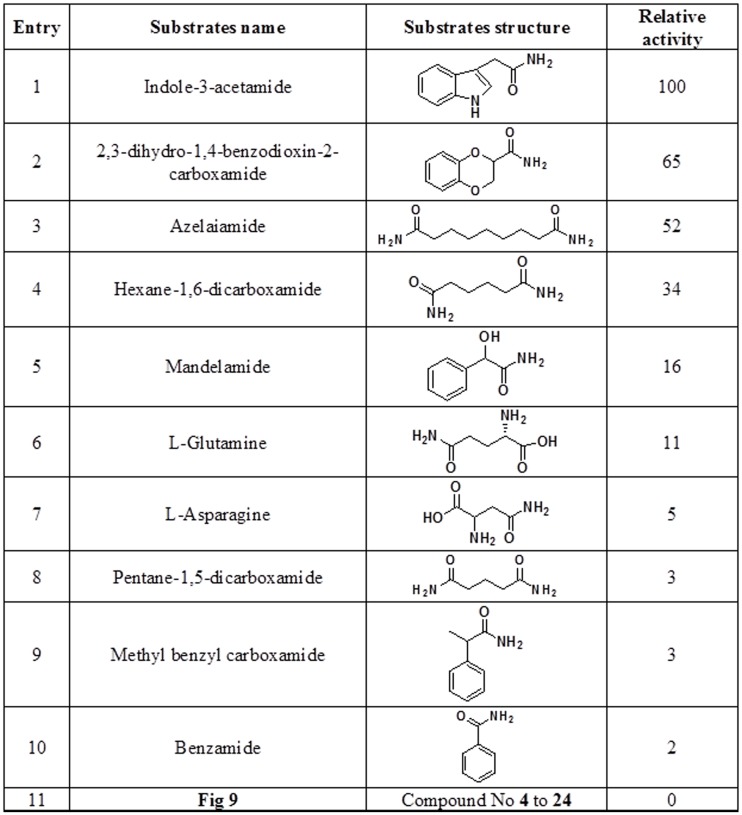
Substrate spectrum of *A*. *faecalis* IaaH. All activities are expressed in per cent relative to activity against indole-3-acetamide, which was taken as 100%. Values are average of experiments done in triplicate.

**Fig 10:**
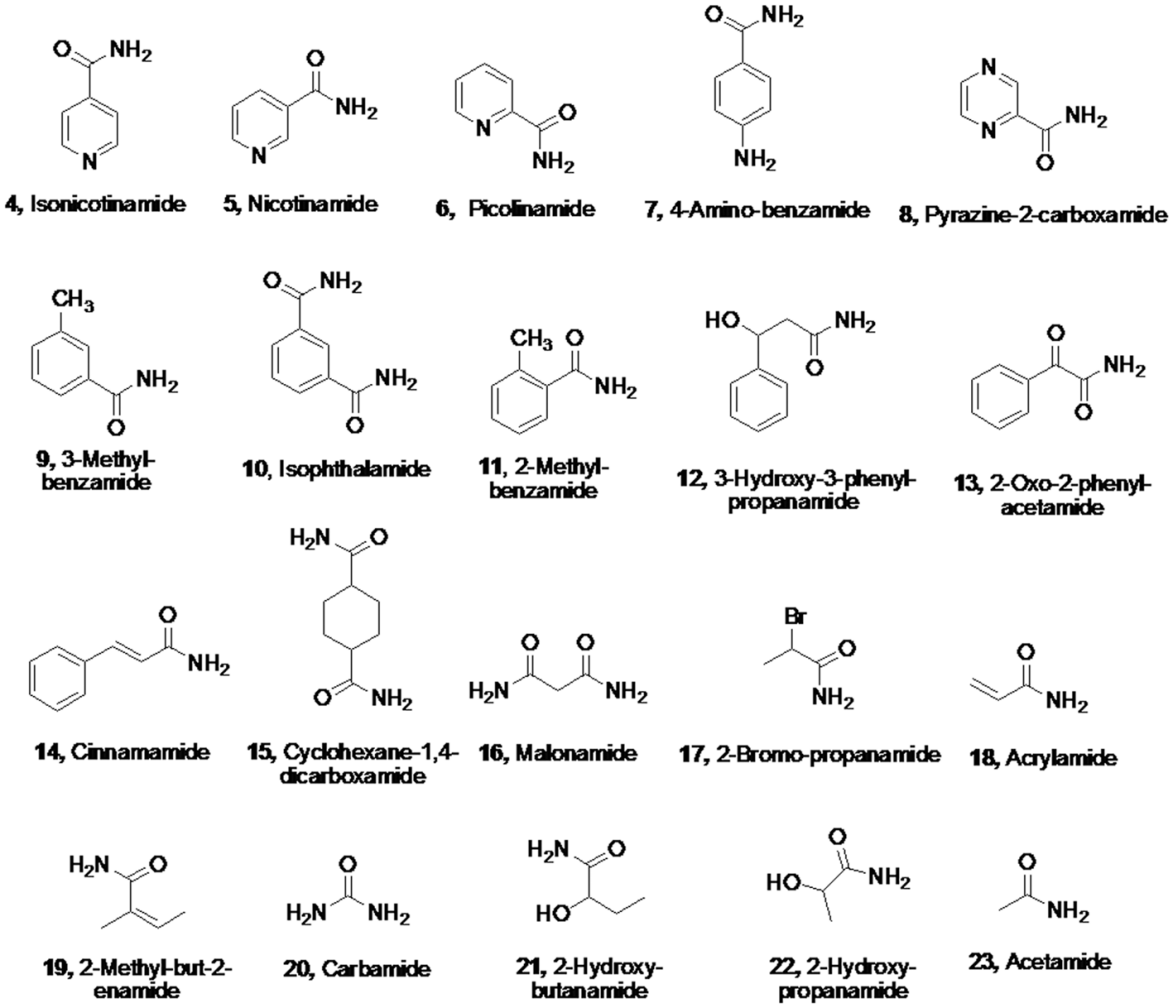
Amides that showed no activity in *A*. *faecalis* catalysed reaction (Entry 11, Fig 9).

To investigate the substrate spectrum for the recombinant enzyme, a variety of potential substrates were tested using the colorimetric assays described in materials and methods section. The results are summarized in Fig 9. The amides, which showed no activity towards the enzyme, have been listed in Fig 10. As expected, IaaH exhibited highest preference for its natural substrate indole-3-acetamide compared to any other substrate tested. The activity of IaaH for indole-3-acetamide was assigned as 100% (Entry 1, Fig 9) and activity for all other substrates was expressed relative to this. It is interesting to note that the activity of IaaH towards 2,3-dihydro-1,4-benzodioxin-2-carboxamide (**2**) was very high (65%; Entry 2, Fig 9). The enzyme displayed good activity towards longer-chain diamides such as azelaiamide, which is C9 diamide (53%, Entry 3, Fig 9) and C6 diamide (32%, Entry 4, Fig 9). The activity fell sharply for chain length lower than C6 with C5 diamide showing only 3% relative activity (Entry 8, Fig 9). The enzyme also hydrolyzed the amino acid amides, but with low activity, e.g. L-glutamine (11%; Entry 6, Fig 9) and L-asparagine (5%; Entry 7, Fig 9). The enzyme showed negligible activity against L-asparagine and no activity against nicotinamide (Entries 6 and 7, Table 2). In comparison, its homolog in *Arabidopsis thaliana*, AtAMI1 showed good activity towards these substrates [31]. Similarly, the enzyme exhibited none or negligible activity against short-chain aliphatic amides, such as acetamide (Entry 11, Fig 9) and simple aromatic amides, such as benzamide (Entry 10, Fig 9). In sharp contrast, its homologs in *Delftia tsuruhatensis* CTCC M 205114 [38], *Geobacillus subterraneus*RL-2a [39], *Paracoccus* sp. M-1 [40], *Pseudonocardia thermophila* [41] exhibited excellent activity towards these substrates. Whereas, benzamide showed low activity, substitution by alkyl, amino, amide or hetero atom resulted in complete loss of activity. Similarly, small chain amides or diamides were inactive (Entry 11, Fig 9). Whereas hexyl-1,6-diamide exhibited good activity, corresponding cyclic isomer cyclohexyl-1,4-diamide was inactive. Thus, the enzyme exhibited excellent activity for bicyclic compounds and longer-chain aliphatic amides, suggesting that the enzyme has a wide pocket in the binding site and hydrophobic interactions are probably important for binding.

In conclusion, we have demonstrated the production of both (***R***) and (***S***)-enantiomers of 2,3-dihydro-1,4-benzodioxin-2-carboxylic acid (**1**), which are important chiral building blocks for doxazosin mesylate, a primary drug used for the benign prostatic hyperplasia and other pharmaceutically important compounds in >99% e.e. by an amidase activity of *Alcaligenes faecalis* subsp. *parafaecalis* catalysed kinetic resolution of benzodioxane-2-carboxamide (**2**). The enzyme exhibited excellent selectivity for (***R*)**-enantiomer with *E* value of >200. Thus, at about 50% conversion, (***R***)-2,3-dihydro-1,4-benzodioxin-2-carboxylic acid (**1**) was obtained in >99% e.e from ***rac***-**2**. The remaining amide had (***S***)-configuration and 99% e.e. The mixture of the (***S***)-amide and (***R***)-acid was separated by aqueous (alkaline)-organic two phase extraction method. The same amidase was able to catalyse, albeit at much lower rate the conversion of (***S***)-amide to (***S***)-acid without loss of e.e. The amidase was identified as indole-3-acetamide hydrolase (IaaH), whose natural role is the production of indole-3-acetic acid from indole-3-acetamide, a phytohormone of auxin class in plants. Incidentally, indole-3-acetamide as the natural substrate for IaaH shared, at least in part a similar bicyclic structure with 2,3-dihydro-1,4-benzodioxin-2-carboxylic acid, which may account for high activity of IaaH towards this un-natural substrate. To the best of our knowledge this is the first application of IaaH in production of industrially important molecule.
